# Probiotics and microbial metabolites maintain barrier and neuromuscular functions and clean protein aggregation to delay disease progression in TDP43 mutation mice

**DOI:** 10.1080/19490976.2024.2363880

**Published:** 2024-06-11

**Authors:** Yongguo Zhang, Yinglin Xia, Jun Sun

**Affiliations:** a Division of Gastroenterology and Hepatology, Department of Medicine, University of Illinois Chicago, Chicago, IL, USA; b Jesse Brown VA Medical Center, Chicago, IL, USA

**Keywords:** Amyotrophic lateral sclerosis, BBB, Claudin-5, ENS, gut-brain axis, inflammation, probiotics, protein aggregation, metabolite, microbiome, SCFA, TDP43, ZO-1

## Abstract

Amyotrophic lateral sclerosis (ALS) is a neuromuscular disease. The ALS mice expressing human mutant of transactive response DNA binding protein of 43 kDa (hmTDP43) showed intestinal dysfunction before neuromuscular symptoms. We hypothesize that restoring the intestinal and microbial homeostasis with a bacterial metabolite or probiotics delays the ALS disease onset. We investigate the pathophysiological changes in the intestine and neurons, intestinal and blood–brain barriers, and inflammation during the ALS progression. We then cultured enteric glial cells (EGCs) isolated from TDP43 mice for mechanistic studies. TDP43 mice had significantly decreased intestinal mobility, increased permeability, and weakened muscle, compared with the age-matched wild-type mice. We observed increased hmTDP43 and Glial fibrillary acidic protein (GFAP), and decreased expression of α-smooth muscle actin (α-SMA), tight junction proteins (ZO-1 and Claudin-5) in the colon, spinal cord, and brain in TDP43 mice. TDP43 mice had reduced Butyryl-coenzyme A CoA transferase, decreased butyrate-producing bacteria *Butyrivibrio fibrisolvens*, and increased *Bacteroides fragilis*, compared to the WT mice. Serum inflammation cytokines (IL-6, IL-17, and IFN-γ) and LPS were elevated in TDP43 mice. EGCs from TDP43 mice showed aggregation of hmTDP43 associated with increased GFAP and ionized calcium-binding adaptor molecule (IBA1, a microglia marker). TDP43 mice treated with butyrate or probiotic VSL#3 had significantly increased rotarod time, increased intestinal mobility and decreased permeability, compared to the untreated group. Butyrate or probiotics treatment decreased the expression of GFAP, TDP43, and increased α-SMA, ZO-1, and Claudin-5 in the colon, spinal cord, and brain. Also, butyrate or probiotics treatment enhanced the Butyryl-coenzyme A CoA transferase, *Butyrivibrio fibrisolvens*, and reduced inflammatory cytokines in TDP43 mice. The TDP43 EGCs treated with butyrate or probiotics showed reduced GFAP, IBA1, and TDP43 aggregation. Restoring the intestinal and microbial homeostasis by beneficial bacteria and metabolites provide a potential therapeutic strategy to treat ALS.

## Introduction

ALS^1^ is a neuromuscular disease that causes the progressive death of motor neurons leading to atrophy. Growing evidence reveals that ALS patients have gastrointestinal symptoms: delayed colonic transit time, inflammation, and dysbiosis.^2,3^ These GI symptoms occur at the early stage of ALS.^3^ Microbiome and intestinal homeostasis are known to play essential roles in neurological diseases, such as ALS,^4,5^ Alzheimer’s disease ,^6–8^ and Parkinson’s disease.^7,9,10^ The complex interplay of gastrointestinal–central nervous system communication involves autonomic and enteric nervous systems, neuroendocrine and immune systems. However, the mechanistic studies of intestinal microbiome through the gut-microbiome-neuron axis in ALS are still limited.

Our group is the first to discover the link between intestinal homeostasis and the disease progression in an ALS mouse model with the human SOD1^G93A^ mutation.^11,12^ Our study in human ALS further reveals the dysbiosis and intestinal inflammation.^4^ Reduced butyrate-producing bacteria correlate with various human gut disorders and central nervous system disorders. Along these lines, ALS SOD1^G93A^ mice had damaged intestinal structure and increased intestinal permeability (leaky gut).^11,12^ Remarkably, restoring the intestinal homeostasis by feeding the ALS model mice with a bacterial metabolite butyrate significantly delayed the disease onset and prolonged the life span of ALS mice and restored the function of enteric neuron system (ENS).^13^ Nicotinamide (Vitamin B3) and nicotinate were found to be reduced in SOD1^G93A^ mice.^5^ However, the clinical applications of microbiome and their beneficial metabolites through the gut-microbiome-neuron axis in ALS are in its infancy.

Human mutant of transactive response DNA binding protein of 43 kDa (TDP43) is known to form abnormal aggregation in neurons and glia, which is the defining pathological hallmark of ALS.^14^ Specific ALS medicine Qalsody, also known as Tofersen, was approved to treat the SOD1 mutation in the ALS patients. However, other mutations and sALS also need for better treatment. There is still a dire need to develop novel treatments for ALS and to improve the life quality of ALS patients.

We hypothesize that restoring the intestinal homeostasis with a bacterial product or probiotics delays the neuromuscular dysfunction and disease onset. In the current study, we tested our hypothesis using the mouse model expressing human mutant TDP43 A315T. We used this TDP43 A315T model because it showed intestinal dysfunction before neuromuscular symptoms.^15^ *In vivo*, we investigate the pathophysiological changes in the intestine and neurons, intestinal and blood-brain barriers, and inflammation during the ALS progression. We investigated the protective role of beneficial bacteria and metabolites by feeding the mice with butyrate or probiotics: significantly delayed the disease onset. *In vitro*, we cultured enteric glial cells (EGCs) isolated from TDP43 mice for mechanistic studies. Our study aims to elucidate the critical role of barriers, EGCs, and microbiome in ALS and to provide insights into alternative approaches for the disease management by restoring the intestinal and microbial homeostasis.

## Materials and methods

### Animals

TDP43 (B6.Cg-Tg(Prnp-TARDBP*A315T)95Balo/J, Strain #: 010700) and age-matched WT mice (C57BL6/J, strain #: 000664) were used in this study, TDP43 and WT mice were purchased from Jackson Laboratory (The Jackson Laboratory, Bar Harbor, ME, USA). All mice were housed in specific pathogen-free environments under a controlled condition of 12 h light/12 h dark cycle at 20°C–22°C and 45  ±  5% humidity, with free access to the same food and autoclaved water. All materials involved including cage, bedding, water bottles, cage card holder were autoclaved before housing mice. The mice were housed with the same gender and each cage had no more than 5 mice. All experiments were carried out in strict accordance with the recommendation in the Guide for the Care and Use of Laboratory Animals of the National Institutes of Health. The protocol was approved by the IACUC of University of Illinois Chicago Committee on Animal Resources (ACC 21–023, ACC21–178, ACC 23–149).

### Rotarod test

Motor coordination, endurance, and balance were determined in latency by a rotarod test. Twelve-week TDP43 and age-matched WT mice were trained on a rotarod test 3 days before performance trials at an accelerating speed from 4 to 40 rpm for 300 s using the Rotarod Model LE8205 (Harvard Apparatus, Holliston, MA, USA). For performance trials, the mice were tested using an accelerated speed from 4 to 40 rpm for 300 s for 3 days in 1 week. Each trial day consisted of three tests per mouse with each test separated by at least 5 min.

### Measurement of gastrointestinal transit times

On the test day, 12-week TDP43 and age-matched WT mice were transferred to individual empty plastic cages (devoid of bedding) and were deprived of food, with free access to water. Two hours after food deprivation, mice were then gavaged with 150 μl of Evans blue marker (5% Evans blue, 5% gum Arabic in drinking water) between 09:00 AM and 09:30 AM local time. After gavage, mice were fed ad libitum. The mice were observed at 5 min-intervals until feces with blue were eliminated (maximum time of observation was 450 min). The time from the end of gavaging to the first blue fecal pellet was measured in minutes and constituted the whole gut transit time.

### Intestinal permeability

Fluorescein isothiocyanate-dextran (Sigma 46,944, Burbank, CA, USA,) diluted in HBSS was gavaged (25 mg/kg mouse) 4 h before sample harvest. Twelve-week TDP43 and age-matched WT mice were anesthetized with avertin; depth of anesthesia was assessed with toe pinch and then blood was collected via cardiac puncture followed by cervical dislocation. Mouse blood samples were collected for intestinal permeability test.^16^

### Butyrate treatment in mice

Age-matched TDP43 mice were divided into groups randomly. The butyrate-treated group mice received sodium butyrate (Sigma-Aldrich 303,410, Milwaukee, WI, USA) at a 2% concentration in autoclaved drinking water starting at 9 weeks of age. Control group mice received autoclaved drinking water without sodium butyrate. All animals were weighted and received detailed clinical examination, which included appearance, movement and behavior patterns, skin and hair conditions, eyes and mucous membranes, respiration and excreta. The mice (12-week-old) were sacrificed under anesthesia by the end of the 3-week butyrate treatment or the time reaching humanely criteria.

### Probiotic treatment in mice

Age-matched TDP43 mice were divided into groups randomly. Mice were daily gavaged with probiotics VSL#3 (Alfasigma USA, Inc. Covington, LA, USA) 1X10^9^ CFU in 0.1 ml of HBSS or an equal volume of HBSS starting at 9 weeks of age. All animals were weighted and received detailed clinical examination, which included appearance, movement and behavior patterns, skin and hair conditions, eyes and mucous membranes, respiration and excreta. The treatment is three-week long. The 12-week-old mice were sacrificed under anesthesia by the end of the probiotics treatment or the time reaching humanely criteria.

### Immunofluorescence

The colonic, spinal cord and brain tissues were freshly isolated and embedded in paraffin wax after fixation with 10% neutral buffered formalin. Immunofluorescence was performed on paraffin-embedded sections (5 μm). After preparation of the slides as described previously,^17^ tissue samples were incubated with anti-GFAP (Cell Signaling, 3670, Danvers, MA, USA), anti-α-SMA (Abcam, ab5694, Waltham, MA, USA), anti-ZO-1 (Invitrogen, 33–9100, Carlsbad, CA, USA), anti-Claudin-5 (Invitrogen, 35–2500, Carlsbad, CA, USA) at 4°C overnight. Samples were then incubated with goat anti-rabbit Alexa Fluor 488 (Invitrogen, A-11008, Carlsbad, CA, USA), or goat anti-mouse Alexa Fluor 488 (Invitrogen, A-11001, Carlsbad, CA, USA) and DAPI (Invitrogen, D1306, Carlsbad, CA, USA) for 1 h at room temperature. Tissues were mounted with SlowFade (Invitrogen, s2828, Carlsbad, CA, USA), followed by a coverslip, and the edges were sealed to prevent drying. Specimens were examined with a Zeiss laser scanning microscope LSM 710 (Carl Zeiss Inc., Oberkochen, Germany). Fluorescence intensity was determined by using ImageJ software. This method determines the corrected total fluorescence by subtracting out background signal, which is useful for comparing the fluorescence intensity between cells or regions.

### Immunohistochemistry (IHC)

After preparation of the slides, antigen retrieval was achieved by incubating the slides for 15 min in hot preheated sodium citrate (pH 6.0) buffer followed by 30 min of cooling at room temperature. Endogenous peroxidases were quenched by incubating the slides in 3% hydrogen peroxide for 10 min, followed by three rinses with HBSS, and incubation for 1 h in 3% BSA + 1% goat serum in HBSS to reduce nonspecific background. Primary antibodies anti-FLAG (Sigma, F3165, Burbank, CA, USA) were applied for overnight in a cold room. After three rinses with HBSS, the slides were incubated in secondary antibody (1:100, Jackson ImmunoResearch Laboratories, 115-065-174, West Grove, PA, USA) for 1 h at room temperature. After washing with HBSS for 10 min, the slides were incubated with vectastain ABC reagent (Vector Laboratories, PK-6100, Burlingame, CA 94,010, USA) for 1 h. After washing with HBSS for 5 min, color development was achieved by applying a peroxidase substrate kit (Vector Laboratories, SK-4800, Burlingame, CA 94,010) for 2 to 5 min, depending on the primary antibody. The duration of peroxidase substrate incubation was determined through pilot experiments and was then held constant for all of the slides. After washing in distilled water, the sections were counterstained with hematoxylin (Leica 3,801,570, Wetzlar, Germany), dehydrated through ethanol and xylene, and cover‐slipped using a permount (Fisher Scientific, SP15–100, MA, USA). The semi-quantitative analysis of IHC staining was performed using software ImageJ Fiji.

### Western blot analysis and antibodies

Twelve-week TDP43 and age-matched WT mice colonic epithelial cells were collected by scraping the tissue from the colon of the mouse, including the proximal and distal regions. Spinal cord was harvested and minced into tiny pieces by using scissors. The collected tissues were sonicated in lysis buffer (10 mM Tris, pH 7.4, 150 mM NaCl, 1 mM EDTA, 1 mM EGTA, pH 8.0, 1% Triton X-100) with 0.2 mM sodium ortho-vanadate, and protease inhibitor cocktail. The protein concentration was measured using the BioRad Reagent (BioRad, Hercules, CA, USA) and then sonicated. Equal amounts of protein were separated by SDS-polyacrylamide gel electrophoresis, transferred to nitrocellulose, and immunoblotted with primary antibodies. The following antibodies were used: anti-FLAG (Sigma, F3165, Burbank, CA, USA), anti-GFAP (Cell Signaling, 3670, Danvers, MA, USA), anti-α-SMA (Abcam, ab5694, Waltham, MA, USA), anti-ZO-1 (Invitrogen, 33–9100, Carlsbad, CA, USA), anti-Claudin-5 (Invitrogen, 35–2500, Carlsbad, CA, USA) or anti-β-actin (Sigma-Aldrich, A5316, St. Louis, MO, USA) antibodies and were visualized by ECL (Thermo Fisher Scientific 32,106, Waltham, MA, USA). Membranes that were probed with more than one antibody were stripped before re-probing. The software Quantity One has been used for the quantification of the western blot bands. Briefly, the “rectangular tool” was first selected to measure the background and the bands of western blots one by one. All the values of “density” and “volume” after measurement were transferred to an excel file. With the subtraction of background measurement, the “density” values for each band on the western blot were calculated.

### Isolation of enteric glial cells (EGCs)

EGCs in mice colon were isolated as previously described with minor modification.^18–20^ Briefly, the colon section was flushed with HBSS until all fecal matter removed into a separate waste container. To remove the Longitudinal Muscle/Myenteric Plexus (LMMP), colon was cut into small segments, approximately 2–4 cm. The gastrointestinal tube was pinned to the rod to prevent the gastrointestinal tract from rotating around rod using the thumb. Bits of mesentery still attached to the gastrointestinal tract were removed using a forceps, followed by creating a gap in the longitudinal muscle by gently rubbing the edge of the forceps along the entire line. From the top of the gap in the longitudinal muscle to the entire strip along the mesentery attachment point, longitudinal muscle was teased away from the circular muscle by applying very light pressure with a cotton swab wetted with HBSS. The LMMP from each of the segments will be easily detached from the remainder of the gastrointestinal tube. The collected LMMP was rinsed three times with PBS containing penicillin (100 U/ml) and streptomycin (100 ug/ml) to remove biological contamination. The rinsed LMMP segments were transferred to the digestion solution and minced into tiny pieces by using scissors and incubated for 30 min with shaking every 10 min at 37°C. The digested LMMP was spined for 5 min at 1000 rpm in a centrifuge cooled to 4°C. After spinning, the digested LMMP was transferred to 0.05% Trypsin for 7 min digestion at 37°C with shaking two times, followed by spinning for 5 min at 1000 rpm in 4°C. The cell pellet was resuspended with 10 ml of complete neuron media, followed by filtering using 100 µm sterile cell strainer and spinning at 1000 rpm for 5 min in 4°C. The collected cell pellet was resuspended in 1.2 ml of complete neuron media and mixed by pipetting slowly and gently until no chunks remained. For the 12-well PDL-laminin coated plate, 100 µL of the cell suspension was added to each well with 900 μl of complete neuron media and incubated in 37°C incubator with 5% CO_2_. Every 2 days, half of the old complete neuron media was replaced with fresh one.

### EGCs treated with butyrate or probiotics VSL#3

Isolated EGCs (3 × 10^5^) were seeded to six-well plates. On day 10, EGCs were treated with butyrate (2 mM) or probiotics VSL#3 (1X10^7^ CFU) in the 1 ml culture medium and incubated in 37°C incubator with 5% CO_2_. Every 12 h, the medium was replaced with fresh culture medium containing butyrate (2 mM) or VSL#3 (1X10^7^ CFU). VSL#3 contains anaerobic bacteria. To keep alive of these strains, the treated cells were incubated in 37°C incubator with 5% CO_2_, which provided Anaerobic environment to some extent. Further, we replaced the old medium with fresh culture medium containing VSL3# every 12 h. Post 48-h treatment, the EGCs were subjected to immunofluorescence staining.

### Real-time PCR measurement of bacterial DNA

DNA was extracted from 12-week TDP43 and age-matched WT mice fecal samples using EZNA Stool DNA Kit (Omega Bio-tek, Inc. D4015–01, Norcross, CA, USA). The quantitative real-time PCR was conducted using the CFX96 Real-time PCR detection system (Bio-Rad Laboratories, Hercules, CA, USA) and iTaq^TM^ Universal SYBR green supermix (Bio-Rad Laboratories 1,725,121, Hercules, CA, USA) according to the manufacturer’s directions. All expression levels were normalized to universal bacteria levels of the same sample. Percent expression was calculated as the ratio of the normalized value of each sample to that of the corresponding untreated control cells. All real-time PCR reactions were performed in triplicate. Primer sequences were designed using Primer-BLAST or were obtained from Primer Bank primer pairs listed in Table 1.

**Table 1.** Real-time PCR Primers.

Primers Name	Sequence
Butyryl-CoA transferase F	5’-GCIGAICATTTCACITGGAAYWSITGGCAYATG-3’
Butyryl-CoA transferase R	5’-CCTGCCTTTGCAATRTCIACRAANGC-3’
*Butyrivibrio fibrisolvens F*	5’-CTAACACATGCAAGTCGAACG-3’
*Butyrivibrio fibrisolvens R*	5’-CCGTGTCTCAGTCCCAATG-3’
*Bacteroides fragilis F*	5′-GGCGCACGGGTG-AGTAACA-3′
*Bacteroides fragilis R*	5′-CAATATTCCTC ACTGCTGC-3′
Universal bacteria F	5’-TCCTACGGGAGGCAGCAGT-3’
Universal bacteria R	5’-GGACTACCAGGGTATCTAATCCTGTT-3’

### Multiplex ELISA assay

The 12-week TDP43 and age-matched WT mice blood samples were collected by cardiac puncture and placed in tubes containing EDTA (10 mg/mL). Mouse cytokines were measured using a Cytokine & Chemokine Convenience 26-Plex Mouse ProcartaPlex™ Panel 1 (Invitrogen, EPXR260 -26,088-90, Carlsbad, CA, USA) according to the manufacturer’s instructions. Briefly, beads of defined spectral properties were conjugated to protein-specific capture antibodies and added along with samples (including standards of known protein concentration, control samples, and test samples) into the wells of a filter-bottom microplate, where proteins bound to the capture antibodies over the course of a 2-h incubation. After washing the beads, protein-specific biotinylated detector antibodies were added and incubated with the beads for 1 h. After removal of excess biotinylated detector antibodies, the streptavidin-conjugated fluorescent protein R-phycoerythrin was added and allowed to incubate for 30 min. After washing to remove unbound streptavidin – R-phycoerythrin, the beads were analyzed with the Luminex detection system (Bio-rad, Bio-Plex 200 Systems, Hercules, CA, USA).

### Serum LPS detection

LPS in serum samples was measured with limulus amebocyte lysate chromogenic end point assays (Hycult Biotech, HIT302, Plymouth, PA, USA) according to the manufacturer’s indications. The 12-week TDP43 and age-matched WT mice serum samples were diluted 1:4 with endotoxin-free water and then heated at 75°C for 5 min on a warm plate to denature the protein before the reaction. A standard curve was generated and used to calculate the concentrations, which were expressed as EU/mL, in the serum samples.

### Statistical analysis

All data were expressed as the mean ± SD. All statistical tests were 2-sided. All *p*-values <0.05 were considered statistically significant. Based on data distributions, the differences between samples were analyzed using Welch’s *t*-test for two groups and two-way ANOVA for more than two groups as appropriate, respectively. Statistical analyses were performed using GraphPad Prism 8 (GraphPad, Inc., San Diego, CA., USA).

## Results

### Slower intestinal motility and augmented intestinal permeability in TDP43 mice

**Figure 1.**
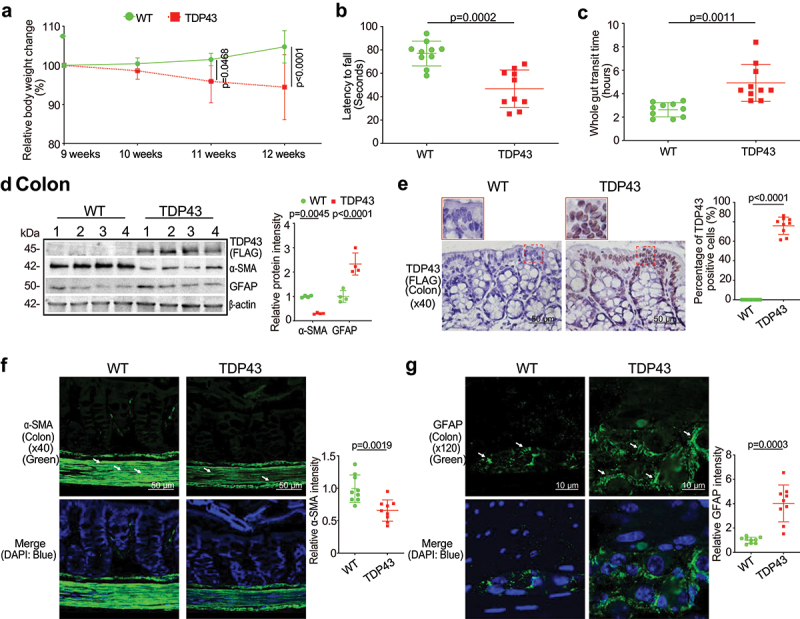
TDP43 mutant mice have less body weight, decreased rotarod test time, slowed intestinal mobility, and altered enteric neuromuscular structure, compared with the age-matched WT mice.

TDP43 mutant mice showed pathological changes, compared with age-matched WT mice at baseline. TDP43 mice started to lose body weight from the age of 11 weeks, compared to WT mice (Figure 1(a)). The hallmark of ALS patients is gradually losing strength in their muscles, which can limit movement. We examined the neuromuscular activity performance of the TDP43 and age-matched WT mice on an accelerating rotarod tests.^13^ TDP43 mice were subjected to the trial on the accelerating spindle (4 to 40 rpm) for 300 s. Latency to fall was recorded when the mouse fell from the rod. Each mouse was tested in 4 trials per day for 2 consecutive days. TDP43 mice had significantly reduced rotarod test time, an indicator of weakened muscle function in TDP43 mice, compared to WT mice at the age of 12 weeks (Figure 1(b)). To study the intestinal function of TDP43 mutant mice, we assessed the transit time of TDP43 mutant and age-matched WT mice, using a whole gut motility assay.^13^ We found significantly increased gut transit time in TDP43 mutant mice, compared with age-matched WT mice (Figure 1(c)), suggesting slower intestinal mobility in TDP43 mice.

(a) TDP43 mutant mice started to show weight loss from the age of 11 weeks compared to WT mice. Each data point represents the average body weight. Data are expressed as mean ± SD. n = 10, two-way ANOVA test. (b) TDP43 mice had significantly reduced rotarod test time compared to WT mice. At the age of 12 weeks, the mice were subjected to the trial on the accelerating spindle from 4 to 40 rpm for 300 s. Latency to fall was recorded when the mouse fell from the rod. Each mouse was tested in 4 trials per day for 2 consecutive days. The mean times for 8 trials of the tests were calculated for each mouse. Data are expressed as mean ± SD. n = 10, Welch’s *t*-test. (c) TDP43 mutant mice had significantly increased gut transit time at 12 weeks compared to WT mice. In age-matched WT and TDP43 mutant mice, intestinal mobility was tested using Evans blue marker (5% Evans blue, 5% gum Arabic in drinking water). Data are expressed as mean ± SD. n = 10, Welch’s *t-*test. (d) At the age of 12 weeks, the expression of α-SMA protein decreased while the expression of GFAP protein increased in intestines of TDP43 mice compared to those in WT mice in western Blotting (WB). Data are shown as mean ± SD. n = 4 per group, Welch’s *t-*test. (e) Human TDP43 protein (FLAG) was observed in intestinal TDP43 mutant mice compared to the age-matched WT mice by IHC staining. Mouse or rabbit IgG was used as the negative experimental control in all the staining. The percentage of positive TDP43 (FLAG) was quantified with ImageJ IHC profiler by counting 3 images for each sample. Data are shown as mean ± SD, n = 3 per group, Welch’s *t*-test. (f) α-SMA expression decreased in intestinal TDP43 mutant mice compared with WT mice, as determined by IF staining. The relative fluorescence intensity was quantified with ImageJ by counting 3 images for each sample. Data are shown as mean ± SD, n = 3 per group, Welch’s *t*-test. (g) GFAP expression increased in the intestines of the TDP43 mutant mice compared to the WT mice in IF staining. Data are shown as mean ± SD, n = 3 per group, Welch’s *t*-test. All *p-*values are shown in the figures.

### Altered enteric neuromuscular markers in the TDP43 mice

Gastrointestinal motor dysfunction occurred in patients with ALS with delayed colonic motility and delayed gastric emptying times.^21^ The gastrointestinal motility is controlled by contractility of smooth muscles of the gastrointestinal tract, ENS, autonomic nervous system and some hormones.^22,23^ The decreased intestinal mobility in TDP43 mice implicated altered enteric nervous cells and intestinal smooth muscle. We then examined the expression of the intestinal smooth muscle marker, α-SMA.^24,25^ There was a significant reduction of α-SMA at the protein level in the intestines of TDP43 mice by western blots (WB) (Figure 1(d)). GFAP is expressed in the glia cells of the ENS, increased in the ENS, spinal cord, brain, and serum of ALS patients, served as a biomarker in ALS.^26,27^ We also found the enhanced GFAP expression in the colon of TDP43 mice by western blots (Figure 1(d)). Interestingly, IHC of TDP 43 showed the significantly enhanced hmTDP43 in the epithelial cells in the colon of TDP43 mice (Figure 1(d)). Immune fluorescence (IF) staining further showed the reduced α-SMA (Figure 1(f)) and enhanced GFAP in the colon of TDP43 mice (Figure 1(g)). Taken together, these data suggested that the expression of hmTDP43 protein in the intestine is correlated with a significantly reduced muscle strength, decreased enteric neuromuscular function, and reduced gut motility.

### Augmented intestinal permeability and altered tight junctions (TJs) in the intestine and spinal cord of the TDP43 mice

**Figure 2.**
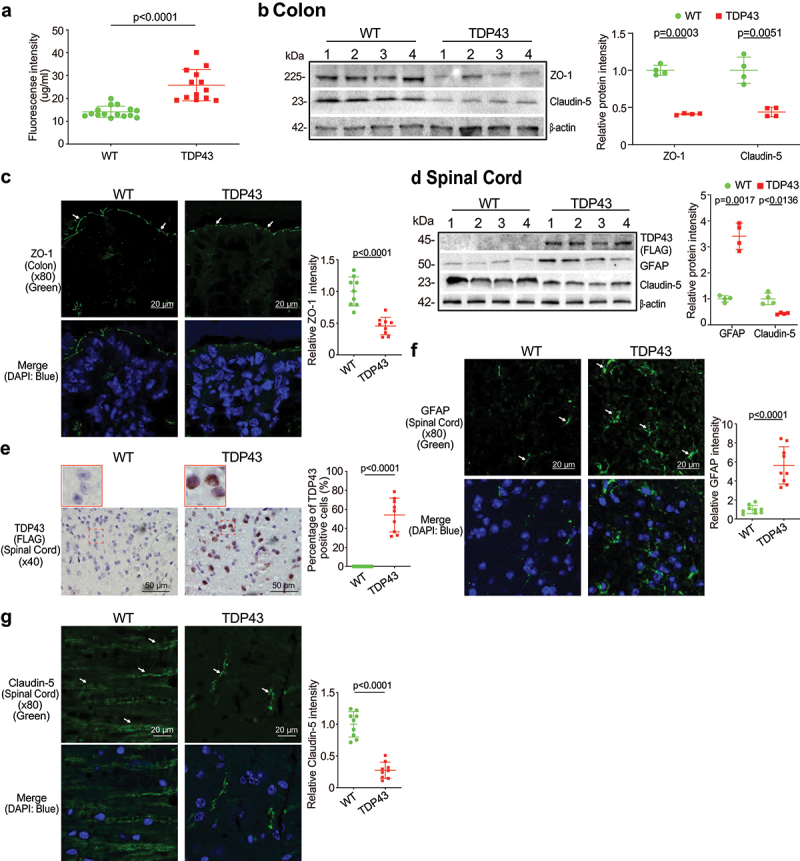
Augmented intestinal permeability and altered tight junctions (TJ) in the intestine, and spinal cord of the TDP43 mice compared with the age-matched WT mice.

To examine the intestinal barrier function through permeability, mice were gavaged with Fluorescein Dextran (molecular weight 4 KD). Four hours later, blood samples were collected for fluorescence intensity measurement. Higher fluorescence intensity indicated increased intestinal permeability. As shown in Figure 2(a), TDP43 mice increased intestinal permeability compared to WT mice. TJ proteins are known to regulate the barrier functions. In the TDP43 mice, we observed significant downregulation of ZO-1 and Claudin-5 at the protein level in the colon of TDP43 mice (Figure 2(b)). Reduced and disorganized ZO-1 was confirmed by the immunostaining of colon in the TDP43 mice (Figure 2(C)).

(a) Intestinal permeability increased in TDP43 mutant mice, compared with WT mice. Data are expressed as the mean ± SD, n = 10, Welch’s *t*-test. (b) ZO-1 and Claudin-5 expression decreased in intestines of TDP43 mice compared to those in WT mice in WB. Data are shown as mean ± SD, n = 4 per group, Welch’s *t-*test. (c) Decreased expression of ZO-1 in the colon of TDP43 mutant mice, compared with WT mice, as determined by IF staining. The relative fluorescence intensity was quantified with ImageJ by counting 3 images for each sample. Data are shown as mean ± SD, n = 3 per group, Welch’s *t-*test. (d) The expression of GFAP protein increased while the expression of Claudin-5 protein decreased in spinal cord of TDP43 mutant mice, compared to WT mice by WB. Data are shown as mean ± SD, n = 3 per group, Welch’s *t-*test. All *p* values are shown in the figures. (e) Human mutant TDP43 protein (FLAG) was observed in the spinal cord of TDP43 mice, compared to the WT mice by IHC staining. The percentage of positive TDP43 (FLAG) was quantified with ImageJ IHC profiler by counting 3 images for each sample. Data are shown as mean ± SD, n = 3 per group, Welch’s *t-*test. (f) GFAP expression increased in the spinal cord of TDP43 mutant mice compared to the WT mice by IF staining. Data are shown as mean ± SD, n = 3 per group, Welch’s *t-*test. (g) Claudin-5 expression decreased in spinal cord of TDP43 mutant mice compared with WT mice, as determined by IF staining. Data are shown as mean ± SD, n = 3 per group, Welch’s *t-*test. All *p-*values are shown in the figures.

Spinal cord degeneration of nerve cells is a hallmark feature of ALS, highlighted in the earliest descriptions of the disease.^28^ Human mutant TDP43 protein (FLAG) was enhanced in TDP43 mutant mouse spinal cord by WB (Figure 2(d)) whereas GFAP expression was increased in the spinal cord of TDP43 mutant mice, compared to that of WT mice by WB (Figure 2(d)). IHC staining of TDP43 indicated its aggregation in the spinal cord of TDP43 mice (Figure 2(e)) IF staining also showed the significant increased GFAP in the spinal cord of TDP43 mice (Figure 2(f)). TJ protein Claudin-5 is a key component of the TJ strand, particularly in spinal cord and brain endothelial cells. The major role of Claudin-5 is to selectively decrease the permeability to ions.^29–31^ Claudin-5 expression was decreased in the spinal cord of TDP43 mutant mice, compared to the WT mice, as determined by WB (Figure 2(d)) and IF staining (Figure 2(g)).

### Increased hmTDP43 and GFAP protein expression and decreased ZO-1 and Claudin-5 in brain of the TDP43 mice

**Figure 3.**
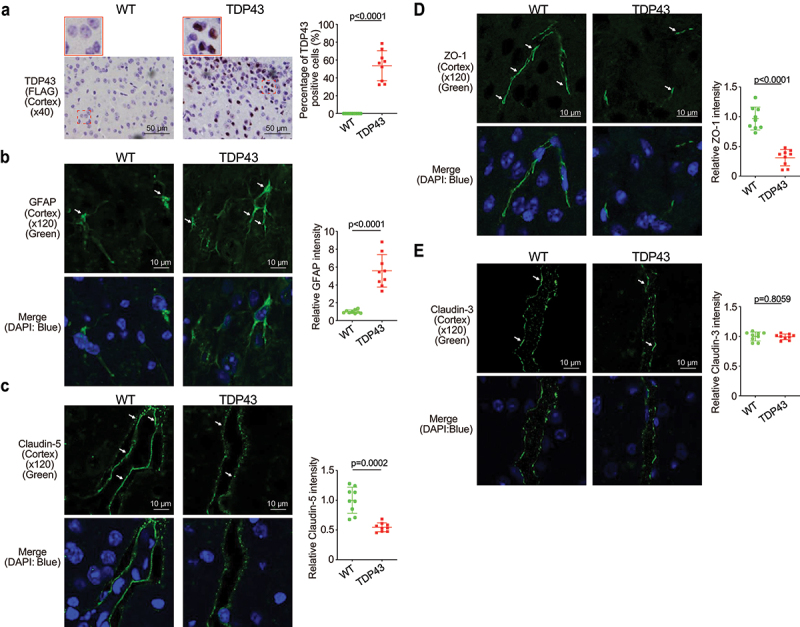
Increased human mutant TDP43 and GFAP protein expression while decreased ZO-1 and Claudin-5 protein expression in the brains of TDP43 mutant mice compared with the age-matched WT mice.

The blood–brain barrier (BBB) is a protective wrapping around the blood vessels of the brain that keeps it safe from toxins and infectious agents.^32,33^ Growing evidence reveals the BBB dysfunction in ALS.^34,35^ The neuron marker GFAP and Claudin-5 and ZO-1 were assessed in the brain of TDP43 mice. Human mutant TDP43 protein (FLAG) was increased in TDP43 mutant mouse brain (Figure 3(a)). GFAP expression was increased in the brain of TDP43 mutant mice compared to that of WT mice by IF staining (Figure 3(b)). Reduced and disorganized Claudin-5 (Figure 3(c)) and ZO-1 (Figure 3(d)) were confirmed by the IF staining in brain of TDP43 mice. In contrast, TJ protein Claudin-3 was very stable in the brain of TDP43 mutant mice and showed unchanged distribution, compared to the WT mice (Figure 3(e)) .

(a) HmTDP43 protein (FLAG) was observed in the brains of TDP43 mice, compared to the WT mice by IHC staining. The percentage of positive TDP43 (FLAG) was quantified with ImageJ IHC profiler by counting 3 images for each sample. Data are shown as mean ± SD, n = 3 per group, Welch’s *t-*test. (b) Increased GFAP expression in the brains of TDP43 mutant mice by IF staining. The relative fluorescence intensity was quantified with ImageJ by counting 3 images for each sample. Data are shown as mean ± SD, n = 3 per group, Welch’s *t-*test. (c) Decreased Claudin-5 in the brains of TDP43 mutant mice compared with WT mice, as determined by IF staining. Data are shown as mean ± SD, n = 3 per group, Welch’s *t-*test. (d) Decreased ZO-1 expression in the brains of TDP43 mutant mice, compared with WT mice. Data are shown as mean ± SD, n = 3 per group, Welch’s *t-*test. (e) Claudin-3 expression was not changed in the brains of the TDP43 mice compared to WT mice, n = 3 per group. All *p-*values are shown in the figures.

### Butyrate treatment reduced hmTDP43 protein expression, enhanced ENS and muscle function, and restored TJs in the intestine of TDP43 mice

**Figure 4.**
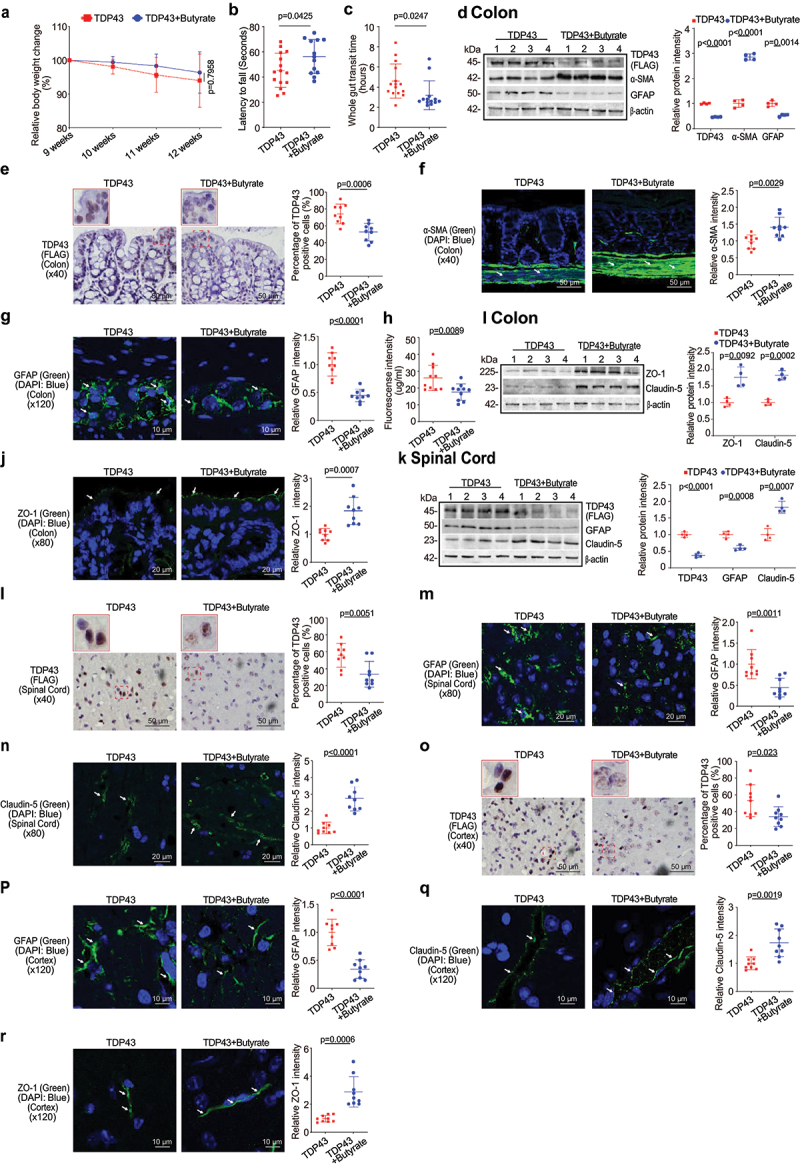
Butyrate treatment led to enhanced ENS and muscle function in the colon, reduced hmTDP43 mutant and GFAP protein expression, increased TJs proteins expression in the colon, spinal cord, and brain of TDP43 mutant mice.

Feeding the SOD1G93A mice with a bacterial metabolite butyrate significantly delayed the disease onset and prolonged the life span of ALS mice.^11^ We then hypothesized that butyrate treatment could inhibit the disease progress in the TDP43 mice. Male or female TDP43 mutant mice were treated with 2% butyrate in the drinking water. The treatment started at 9 weeks of age and ended at 12 weeks of age. The treatment duration is total 3 weeks. Butyrate treated TDP43 mice show less weight loss from the age of 11 weeks, compared to the no-treatment TDP43 mice (Figure 4(a)). TDP43 mice with butyrate treatment showed a significantly longer latency to fall in the rotarod test time, compared to the TDP43 mice without treatment (Figure 4(b)). TDP43 mutant mice with butyrate treatment showed a significantly decreased gut transit time (Figure 4(c)). The butyrate-treated TDP43 mice had reduced hmTDP43 protein and GFAP expression and enhanced α-SMA expression in the colon, compared to non-treated TDP43 mice by WB (Figure 4(d)). Reduced human mutant TDP43 protein was confirmed by the IHC staining of intestines in the TDP43 mice (Figure 4(e)). There was increased expression of α-SMA post butyrate treatment in the TDP43 mice (Figure 4(f)). Butyrate treatment led to decreased GFAP expression in the intestines of TDP43 mutant mice (Figure 4(g)). Intestinal permeability was decreased in TDP43 mutant mice treated with butyrate (Figure 4(h)). We also found the enhanced ZO-1 and Claudin-5 in the colon of TDP43 mice with butyrate treatment by WB (Figure 4(i)) and IF staining (Figure 4(j)). These data suggested that butyrate treatment restored key proteins for enteric neuromuscular and intestinal barrier function at the protein and cellular levels.

(a) Body weight changes of TDP43 mutant mice after butyrate treatment. Male or female TDP43 mutant mice were treated with or without 2% butyrate in the drinking water from 9 weeks to 12 weeks. Butyrate treated TDP43 mutant mice show slight weight loss without statistical significance from the age of 11 weeks, compared to the TDP43 mice without treatment. Data are expressed as mean ± SD. n = 13-15, two-way ANOVA test. (b) TDP43 mutant mice with butyrate treatment showed a significantly increased rotarod test time. Data are expressed as mean ± SD. n = 13-15, Welch’s *t*-test. (c) TDP43 mutant mice with butyrate treatment showed a significantly decreased gut transit time. Data are expressed as mean ± SD. n = 13-15, Welch’s *t-*test. (d) The butyrate treated TDP43 mutant mice had reduced hmTDP43 and GFAP, and enhanced α-SMA protein expression in the intestines, compared to non-treated TDP43 mutant mice in WB. Data are shown as mean ± SD, n = 4 per group, Welch’s *t-*test. (e) Butyrate treatment led to decreased hmTDP43 expression in the intestines of TDP43 mutant mice. The percentage of positive TDP43 (FLAG) was quantified with ImageJ IHC profiler by counting 3 images for each sample. Data are shown as mean ± SD, n = 3 per group, Welch’s *t-*test. (f) Butyrate treatment led to increased α-SMA expression in the intestines of TDP43 mutant mice. The relative fluorescence intensity was quantified with ImageJ by counting 3 images for each sample. Data are shown as mean ± SD, n = 3 per group, Welch’s *t-*test. (g) Butyrate treatment led to decreased GFAP expression in the intestines of TDP43 mutant mice. Data are shown as mean ± SD, n = 3 per group, Welch’s *t-*test. (h) Intestinal permeability decreased in TDP43 mutant mice treated with butyrate. Data are expressed as mean ± SD. n = 10, Welch’s *t*-test. (i) The butyrate treated TDP43 mutant mice had enhanced ZO-1 and Claudin-5 protein expression in the intestines compared to non-treated TDP43 mutant mice in WB. Data are shown as mean ± SD, n = 4 per group, Welch’s *t-*test. (j) Increased ZO-1 expression in the colon of TDP43 mutant mice with butyrate treatment in IF. Data are shown as mean ± SD, n = 3 per group, Welch’s *t-*test. (k) Reduced human TDP43 protein expression, decreased GFAP expression and enhanced Claudin-5 expression in the spinal cord of TDP43 mutant mice treated with butyrate in WB. Data are shown as mean ± SD, n = 4, Welch’s *t-*test. (l) Butyrate treatment led to decreased hmTDP43 in the spinal cord of TDP43 mutant mice. Data are shown as mean ± SD, n = 3 per group, Welch’s *t-*test. (m) Butyrate treatment led to decreased GFAP expression in the spinal cord of TDP43 mutant mice by IF. Data are shown as mean ± SD, n = 3 per group, Welch’s *t-*test. (n) Butyrate treatment led to increased Claudin-5 expression in the spinal cord of TDP43 mutant mice, as determined by IF. Data are shown as mean ± SD, n = 3 per group, Welch’s *t-*test. (o) Butyrate treatment led to decreased human TDP43 expression in the brains of TDP43 mice. Data are shown as mean ± SD, n = 3 per group, Welch’s *t-*test. (p) Butyrate treatment led to decreased GFAP expression in the brains of TDP43 mice in IF staining. Data are shown as mean ± SD, n = 3 per group, Welch’s *t-*test. Butyrate treatment led to increased Claudin-5 (q) and ZO-1 (r) expression in the brains of TDP43 mice, as determined in IF staining. Data are shown as mean ± SD, n = 3 per group, Welch’s *t-*test. All *p-*values are shown in the figures.

### Butyrate treatment led to reduced hmTDP43 aggregation and GFAP protein and increased TJs proteins in the spinal cord and brain of TDP43 mice

In the spinal cord of TDP43 mice, we found that butyrate treatment led to reduced hmTDP43 protein expression, decreased GFAP expression and enhanced Claudin-5 expression, compared to the TDP43 mice without treatment (Figure 4(k)). Changes of these proteins were confirmed by the IHC or IF staining of spinal cord in the TDP43 mice treatment or without treatment with butyrate. Butyrate treatment led to decreased human TDP43 (Figure 4(l)) and GFAP (Figure 4(m)) expression in the spinal cord of TDP43 mice by immunostaining staining. Claudin-5 expression was increased in the spinal cord of butyrate-treated TDP43 mice compared to the TDP43 mice without treatment (Figure 4(n)). Butyrate treatment led to decreased expression of human mutant TDP43 (Figure 4(o)) and GFAP (Figure 4(p)) in the brains of TDP43 mice, compared to the TDP43 mice without treatment. Butyrate treatment led to increased Claudin-5 (Figure 4(q)) and ZO-1 (Figure 4(r)) in the brains of TDP43 mice, compared to the TDP43 mice without treatment.

### Probiotics treatment led to enhanced ENS and muscle function in the intestines of TDP43 mice

**Figure 5.**
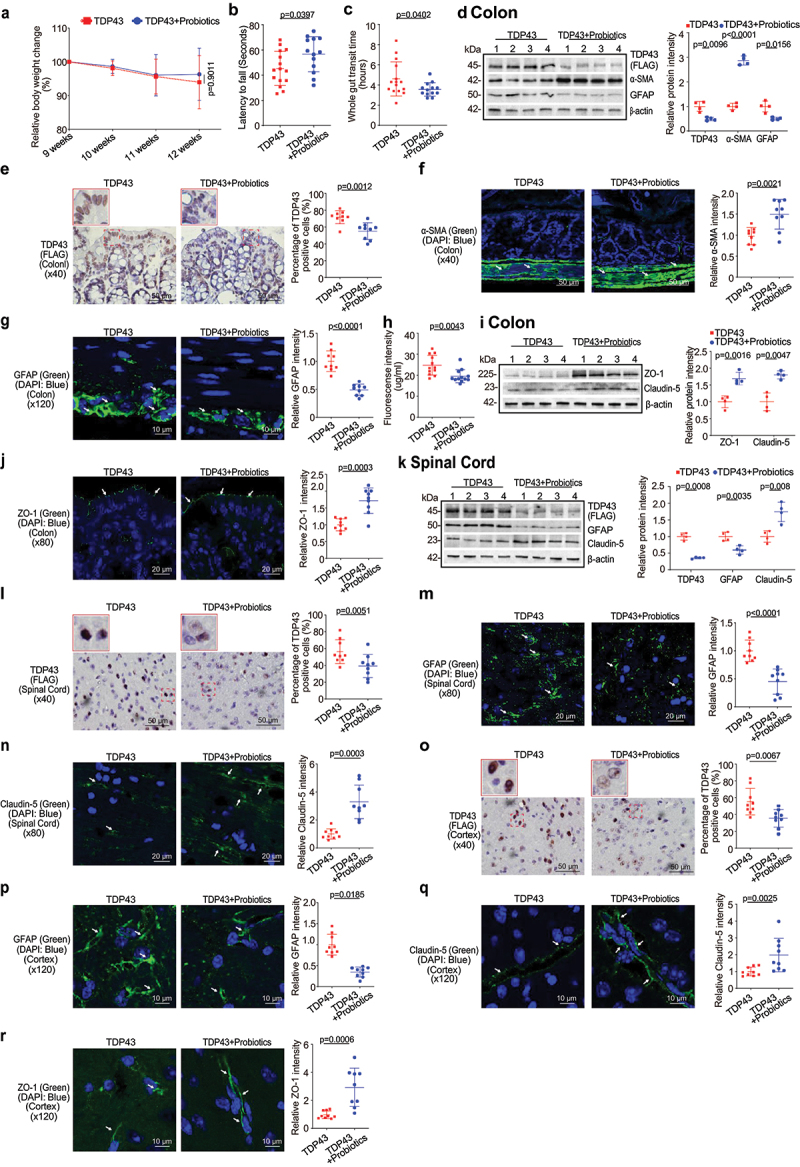
Probiotics treatment led to enhanced ENS and muscle function in the intestines, reduced hmTDP43 and GFAP protein expression, increased TJs proteins expression in the intestine, spinal cord, and brain of TDP43 mutant mice.

Probiotics are live microorganisms and have been proven to have a health effect on hosts.^36,37^ Probiotic mixture VSL#3 contains 8 complimentary strains: *Lactobacillus acidophilus, Lactobacillus plantarum, Lactobacillus paracasei, Lactobacillus helveticus*, *Bifidobacterium breve, Bifidobacterium longum*, *Bifidobacterium infantis*, and *Streptococcus thermophilus*.^38^ Male or female 9-week old TDP43 mice were treated with probiotics (VSL#3, 1X 10^9^ CFU) daily by oral gavage starting for 3 weeks. TDP43 mice with probiotic treatment show less weight loss from the age of 11 weeks (Figure 5(a)). Butyrate treatment led to a significantly longer latency to fall in the rotarod test, compared to the TDP43 mice without treatment (Figure 5(b)). We found that TDP43 mutant mice with VSL#3 treatment showed a significantly decreased intestinal transit time (Figure 5(c)). VSL#3 treatment led to decreased hmTDP43 and GFAP, increased α-SMA expression in the intestines of TDP43 mice (Figure 5(d)). Reduced hmTDP43 was confirmed by IHC staining of intestine in the TDP43 mice (Figure 5(e)). Figure 5(f) showed the significantly increased α-SMA expression in the colon of TDP43 mice with VSL#3 treatment. Also, there was decreased expression of GFAP post probiotics treatment in the intestines of the TDP43 mice (Figure 5(g)). Intestinal permeability was decreased in TDP43 mutant mice with probiotic treatment (Figure 5(h)). Enhanced expression of ZO-1 and Claudin-5 protein was found in the colon of TDP43 mice with probiotics treatment by WB (Figure 5(i)) and IF staining (Figure 5(j)).The data indicate that intestinal TJs have been maintained in TDP43 mice treatment with probiotics.

(a) Body weight changes of TDP43 mutant mice after probiotics treatment. Male or female TDP43 mice were treated with or without probiotics (VSL#3, 1 × 109CFU) daily by oral gavage starting from 9 weeks to 12 weeks. Probiotic treated TDP43 mice showed slight weight loss without a statistical significance at the age of 12 weeks, compared to the no-treatment TDP43 mutant mice. Data are expressed as mean ± SD. n = 13-15, two-way ANOVA test. (b) TDP43 mutant mice with probiotics treatment showed a significantly increased rotarod test time. Data are expressed as mean ± SD. n = 13-15, Welch’s *t*-test. (c) TDP43 mutant mice with probiotics treatment showed a significantly decreased gut transit time. Data are expressed as mean ± SD. n = 13-15, Welch’s *t-*test. (d) The VSL#3 treated TDP43 mutant mice had reduced hm TDP43 and GFAP expression, and enhanced α-SMA in the colon, compared to TDP43 mutant mice without treatment, as determined in WB. Data are shown as mean ± SD, n = 4 per group, Welch’s *t-*test. VSL#3 treatment led to decreased hmTDP43 (e), increased α-SMA expression (f) and decreased GFAP expression (g) in the colon of TDP43 mutant mice. Data are shown as mean ± SD, n = 3 per group, Welch’s *t-*test. (h) Intestinal permeability decreased in TDP43 mutant mice treated with probiotics. Data are expressed as mean ± SD. n = 10, Welch’s *t-*test. (i) Increased ZO-1 and Claudin-5 protein expression in the intestines of TDP43 mutant mice with VSL#3 treatment in WB. Data are shown as mean ± SD, n = 4 per group, Welch’s *t*-test. (j) Increased ZO-1 expression in the intestines of TDP43 mutant mice with probiotics treatment in IF. Data are shown as mean ± SD, n = 3 per group, Welch’s *t-*test. (k) Reduced hmTDP43 and GFAP expression, enhanced Claudin-5 expression in the spinal cord of TDP43 mutant mice treated with probiotics. Data are shown as mean ± SD, n = 4, Welch’s *t-*test. Probiotics treatment led to decreased hmTDP43 (l), decreased GFAP expression (m) and increased Claudin-5 expression (n) in the spinal cord of TDP43 mutant mice, as determined by IHC or IF staining. Data are shown as mean ± SD, n = 3 per group, Welch’s *t-*test. Probiotics treatment led to reduced human TDP43 expression (o), decreased GFAP expression (p), increased Claudin-5 expression (q), and increased ZO-1 expression (r) in the brains of TDP43 mice. Data are shown as mean ± SD, n = 3 per group, Welch’s *t-*test. All *p-*values are shown in the figures.

### Probiotic treatment led to increased TJs proteins expression in the spinal cords and brain of TDP43 mice

Probiotic treatment was able to reduce the hmTDP43 aggregation and GFAP expression in the spinal cord of TDP43 mice (Figures 5(k-m)). TJ Claudin-5 expression was increased in the spinal cord of TDP43 mutant mice with probiotics treatment, compared to the TDP43 mice without treatment, as determined by WB (Figure 5(k)) and IF staining (Figure 5(n)). Probiotic treatment led to decreased expression of hmTDP43 (Figure 5(o)) and GFAP (Figure 5(p)) in the brains of TDP43 mice treated with probiotics. Probiotics treatment also maintained TJ proteins, Claudin-5 (Figure 5(q)) and ZO-1 (Figure 5(r)), in the brains of TDP43 mice.

### Butyrate or probiotics VSL#3 treatment corrected dysbiosis in the TDP43 mice

**Figure 6.**
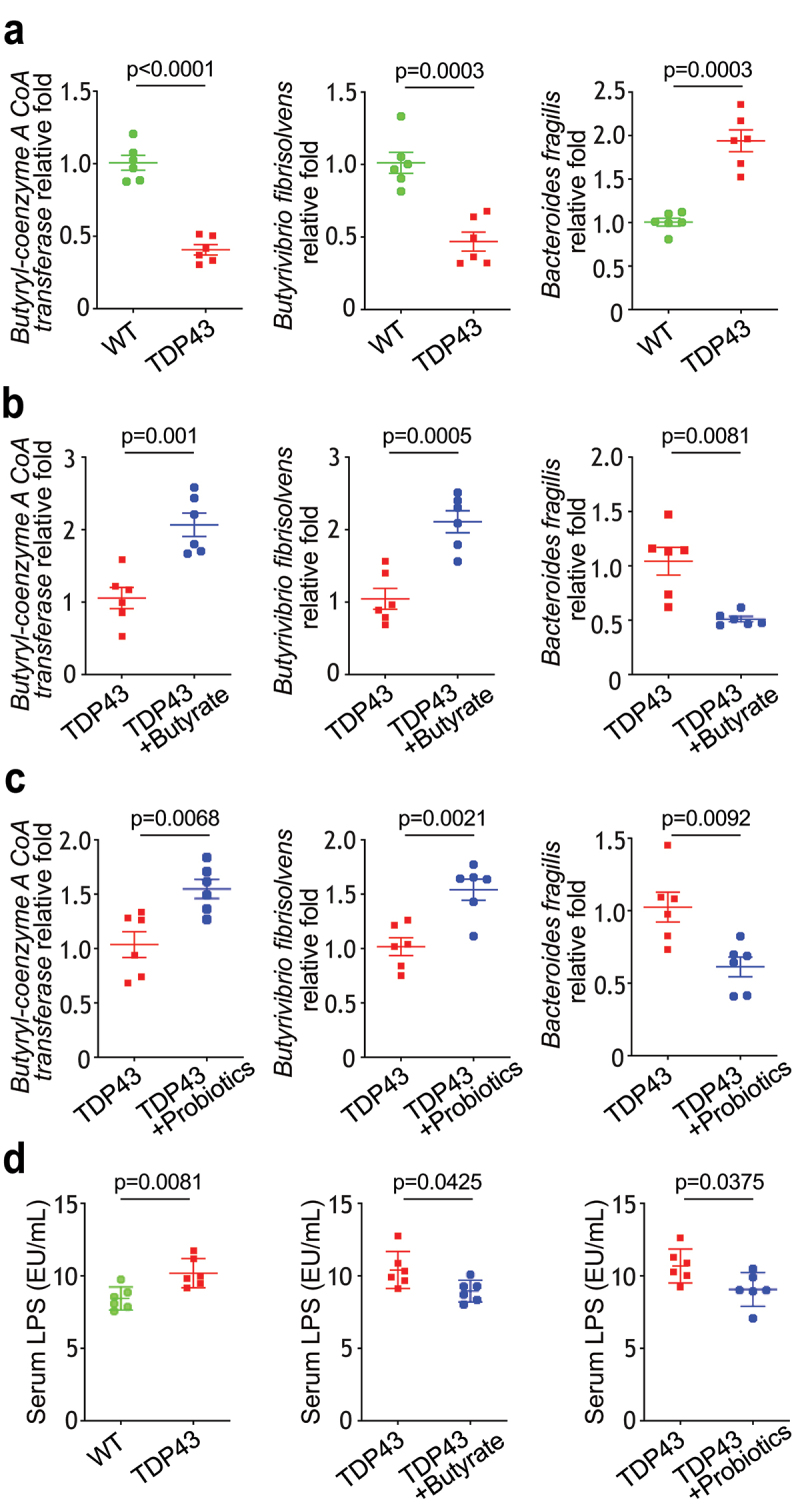
Butyrate or VSL#3 treatment corrected dysbiosis in ALS TDP43 mice, modulating microbiome leads reduced LPS expression in ALS TDP43 mice.

Butyrate can be synthesized via butyryl CoA:acetate CoA transferase, Butyryl-CoA:acetate CoA-transferase transports the CoA component to external acetate, resulting in the release of butyrate and acetyl-CoA.^39,40^ We found that the level of butyryl-CoA transferase (BCoAT) gene was significantly lower in the TDP43 compared with that in the WT mice (Figure 6(a)). In our previous study, SOD1^G93A^ mice intestinal microbial homeostasis was restored via treatment with butyrate.^11^ There was an increase in the level of butyryl-CoA transferase gene in the feces TDP43 mice treated with butyrate (Figure 6(b)) or probiotics VSL#3 (Figure 6(c)). Population of butyrate-producing bacteria *Butyrivibrio fibrisolvens* ^41,42^ was reduced in the TDP43 mice (Figure 6(a)).

(a) TDP43 mice led to dysbiosis and a shift in the bacterial profile. TDP43 mice decreased butyryl-coenzyme A CoA transferase genes, decreased *Butyrivibrio fibrisolvens* and increased Bacteroides fragilis, compared with WT mice. Data are expressed as the mean ± SD. N = 6, Welch’s *t-*test. (b) Butyrate or (c) probiotics treatment increased butyryl-coenzyme A CoA transferase gene, increased *Butyrivibrio fibrisolvens* and decreased *Bacteroides* fragilis in the TDP43 mice treated with butyrate or VSL#3. Data are expressed as the mean ± SD. n = 6, Welch’s *t-*test. (d) LPS were significantly higher in TDP43 mice than that in WT mice, but reduced LPS level was found in TDP43 mice after butyrate or probiotics treatment. Data are expressed as the mean ± SD, n = 6, Welch’s *t-*test. All *p*-values are shown in the figures.

Treatment with butyrate (Figure 6(b)) or probiotics VSL#3 (Figure 6(c)) also led to significant enhancement of *Butyrivibrio fibrisolvens*. *Bacteroides fragilis* is the most common cause of anaerobic infections in humans. Infection due to *Bacteroides fragilis* is usually polymicrobial and results from a disruption in tissue barriers.^43,44^ *Bacteroides fragilis* has higher abundance in TDP43 mice (Figure 6(a)), compared with WT mice, was reduced after butyrate (Figure 6(b)) or probiotics *VSL#3* treatment (Figure 6(c)). Increased serum lipopolysaccharides (LPS), the outer membrane components of gram-negative bacteria, is related with dysbiosis, inflammation, and dysregulated barrier function. We found that butyrate or probiotics *VSL#3* treatment reduced the serum LPS in the TDP mice (Figure 6(d)). Patients with ALS had increased LPS.^11,12,45–50^ We have shown that manipulating the microbiome and reduced LPS in SOD1^G93A^ mice.^50^ Thus, our data suggest the potential to manipulate microbiome in ALS treatment.

### Modulating microbiome leads to reduced serum IL-6, IL-17, and IFN-γ in TDP43 mice

**Figure 7.**
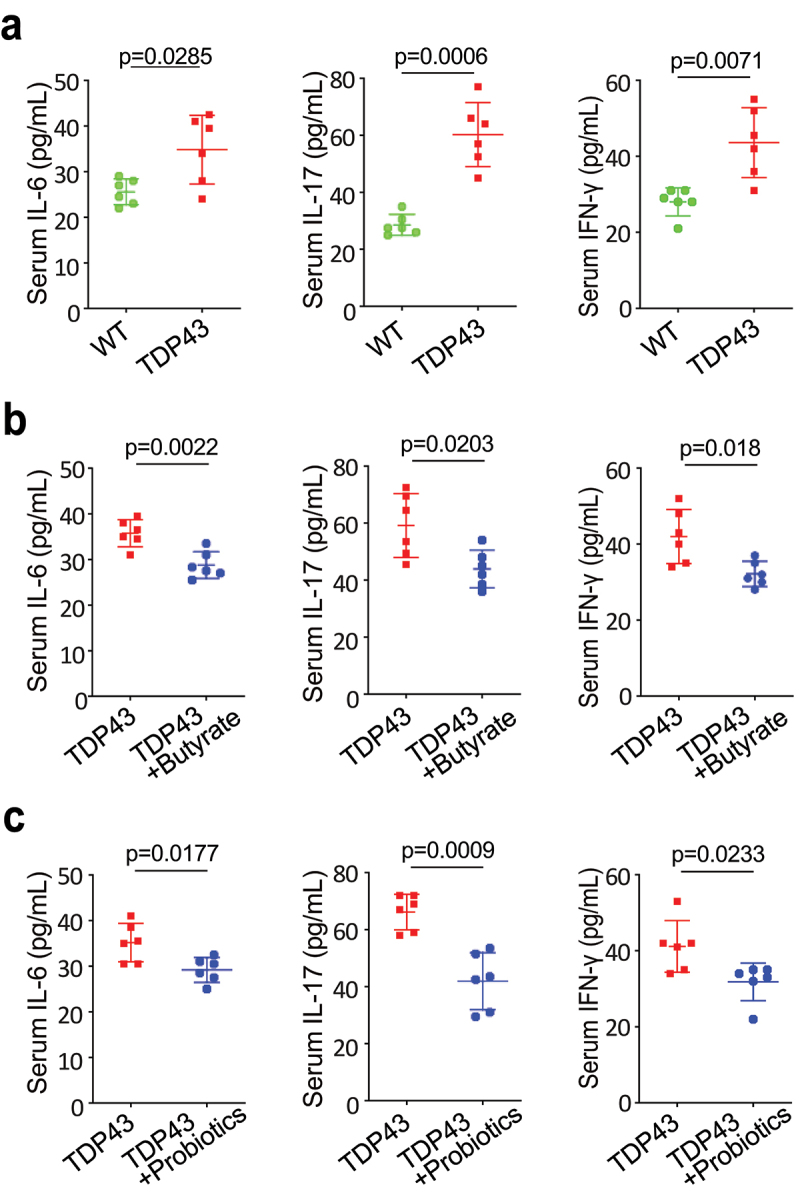
Butyrate or probiotic VSL#3 treatment reduced serum IL-6, IL-17, and IFN-γ expression in TDP43 mice.

Serum inflammatory cytokines were found to be elevated in patients with ALS, compared with healthy controls, and were associated with clinical markers of disease severity.^11,12,45–50^ We have shown that manipulating the microbiome via butyrate treatment decreases inflammatory responses in SOD1^G93A^ mice.^50^ We observed significantly increased levels of sera IL-6, IL-17 and IFN-γ l in the TDP43 mutant mice (Figure 7(a)). Sera IL-6, IL-17 and IFN- γ were significantly lower in the TDP43 mutant mice with butyrate (Figure 7(b)) or probiotics treatment (Figure 7(c)), suggesting that butyrate or probiotics protected TDP43 mutant mice from increased inflammation.

(a) Serum cytokines, IL-6, IL-17, and IFN-γ, were significantly higher in TDP43 mutant mice. Serum samples were collected from TDP43 mutant mice and age-matched WT mice, and then cytokines were detected by a Luminex detection system. Data are expressed as the mean ± SD, n = 6, Welch’s *t-*test. (b) Serum IL-6, IL-17, and IFN-γ were significantly lower in TDP43 mice treated with butyrate. Data are expressed as the mean ± SD, n = 6, Welch’s *t-*test. (c) Serum IL-6, IL-17, and IFN-γ were significantly lower in TDP43 mice treated with VSL#3. Data are expressed as the mean ± SD, n = 6, Welch’s *t* -test. All *p-*values are shown in the figures.

### Microbial metabolite decreased aggregation of hmTDP43 protein, decreased GFAP and IBA1 expression in EGCs with TDP43 mutant in vitro

**Figure 8.**
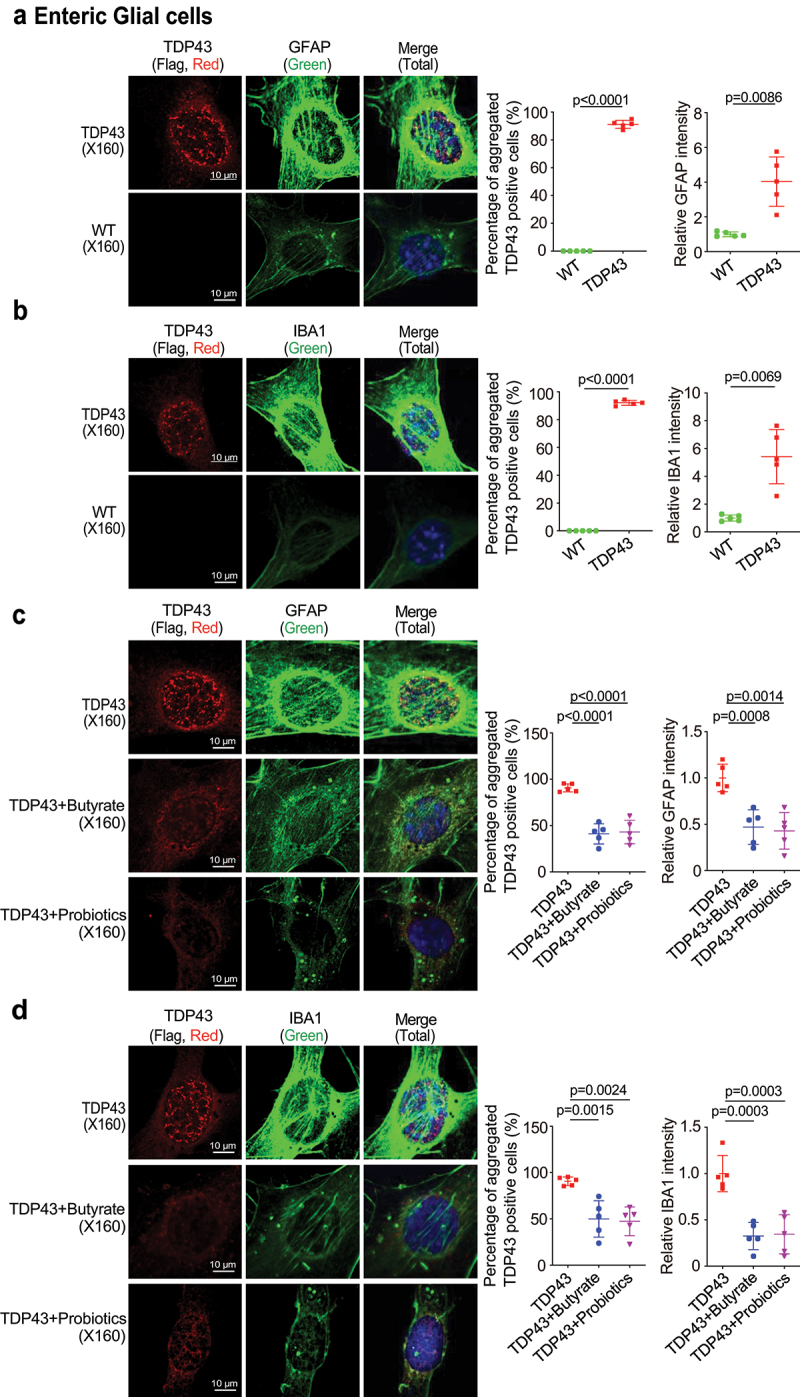
Butyrate or probiotics treatment decreased aggregation of hmTDP43 protein, decreased GFAP and IBA1 expression in EGCs isolated from TDP43 mutant mice.

Human mutant TDP-43 protein aggregation is considered a hallmark of ALS patients’ pathological phenomena.^51–55^ To better understand the mechanism of butyrate or probiotics in improving intestinal and neuromuscular function of TDP43 mice, we isolated EGCs cells from the TDP43 mice (Figure 8(a)). We found hm TDP43 protein aggregates (red) and increased GFAP protein expression (green) in the EGCs isolated from TDP43 mutant mice, compared to the age-matched WT mice (Figure 8(a)). IBA1 (Ionized calcium binding adaptor molecule 1) is a microglia/macrophage-specific calcium-binding protein. IBA1 is upregulated when microglia are activated.^56–58^ IBA1 increased in the ENS, spinal cord, brain of ALS patients.^59–61^ Thus, we examined IBA1 and found the increased IBA1 protein in the isolated TDP43 mutant EGCs, compared to those from the WT mice (Figure 8(b)). We then examined whether butyrate or probiotics treatment could reduce the protein aggregation of hmTDP43, and expression levels of GFAP and IBA1 in the EGCs cells. The EGCs cells isolated from TDP43 mutant mice (called TDP43 EGCs) were treated with butyrate or probiotics. Isolated EGCs were treated with butyrate (2 mM, 48 h) or probiotics VSL#3 (1X10^7^ CFU, 48 h) in the culture medium. There was significant reduction in hmTDP43 aggregation and GFAP protein expression (Figure 8(c)) . Butyrate or probiotics treatment also reduced IBA1 expression (green), while reducing hmTDP43 (red) in TDP43 mutant EGCs (Figure 8(d)).

(a) Aggregated hmTDP43 protein and increased GFAP were observed in the TDP43 EGCs. TDP43 protein aggregates were quantified with AggreCount. Data are shown as mean ± SD, n = 5, Welch’s *t-*test. (b) Aggregated human TDP43 protein and increased IBA1 protein expression were observed in the EGCs from TDP43 mice, compared with the WT EGCs. Data are shown as mean ± SD, n = 5, Welch’s *t-*test. (c) Butyrate or probiotics treatment reduced hmTDP43 aggregation and decreased GFAP expression in cultured TDP43 EGCs. Data are expressed as mean ± SD, n = 5, one-way ANONA test. (d) Butyrate or probiotics treatment reduced hmTDP43 aggregation and decreased IBA1 in EGCs with hmTDP43. Data are expressed as mean ± SD, n = 5, one-way ANONA test. *p-*values are shown in the figures.

## Discussion

In the current study, we provide the first evidence that beneficial metabolite butyrate and probiotic VSL#3 are able to enhance the intestinal mobility, barrier function, and reduce hmTDP43 aggregation through the gut-microbiome-neuron axis. TDP43 mutant mice without treatment had increased hmTDP43 and GFAP, and decreased expression of α-Smooth muscle actin (α-SMA), tight junction proteins (ZO-1 and Claudin-5) in the colon, spinal cord, and brain of TDP43 mice. Serum inflammation cytokines and LPS were elevated in TDP43 mice. TDP43 EGCs showed aggregation of hmTDP43 protein associated with increased expression of GFAP and IBA1. We treated 9-weeks TDP43 mice with butyrate and probiotics (VSL#3) to investigate the effect of modulating microbiome on neuromuscular function. Three- weeks posttreatment, treated TDP43 mice had significantly increased rotarod time, increased intestinal mobility, and enhanced barrier function, compared to the untreated group mice. Butyrate or probiotics treatment decreased the expression of TDP43 and GFAP, and increased expression of α-SMA, ZO-1, and Claudin-5 in the colon, spinal cord, and brain. Mechanistically, we report that butyrate or probiotics reduced hmTDP43 aggregation and decreased expression of GFAP and IBA1 in TDP43 EGCs.

The specialized micro-environment of the central nervous system is maintained by BBB.^62^ The state of brain capillaries and their polarized microvascular endothelial cells are responsible for BBB structure and functional integrity, by possessing TJs.^63^ In the current study, we showed increased expression of TJ protein ZO-1 and Claudin-5 not only in the colon post butyrate or probiotic treatment, but also in the spinal cord and brain of the TDP43 mutation mice. Occludin, Claudin, junctional adhesion molecules, and ZO-1 are the main elements of intercellular TJ proteins. Control paracellular passage of substrates across the BBB is the main function of these proteins. Any damage to or detachment of this protein from its counterparts may result in enhancement of permeability. In SOD1^G93A^ mice, a disrupted blood-spinal cord barrier as well as decreased levels of TJ protein Claudin-5 was detected.^31,64^ Probiotic mixture VSL#3 was able to improve nervous systemic disorders and related brain mechanisms through the gut-brain in rats.^65^ Our data have demonstrated the protective role of probiotics in maintaining Claudin-5 and ZO-1 in the brain of TDP43 mice. Obtaining knowledge of BBB regulated by bacteria or bacterial metabolites in ALS will provide a picture of how the TJ proteins contribute to the changes of the CNS. Furthermore, our data have indicated the new role of Claudin-5 manipulated by the inflammation and dysbiosis in the colon, spinal cord, and brain. It will support the discovery of novel therapeutic strategies for managing ALS through the gut-microbiome-brain axis.

There are two forms of ALS: sporadic (sALS) and familial (fALS).^1^ The majority of ALS cases are sporadic and only about 20% of familial forms. Even in families with genetic predisposition, there is significant phenotypic variability, suggesting that ALS onset may be triggered by a combination of genetic factors, environmental factors (*e.g*., military service and deployments, exposures),^66^ lifestyle changes (*e.g*., cigarette smoke and dietary), and gut microbiome alteration. For reasons yet unknown, Veterans are twice as likely to be diagnosed with ALS as the general population.^66^ The Department of Veterans Affairs has recognized ALS as a service-connected disease. Although the initial factors are different, the sequential symptoms of fALS and sALS are similar. Both SOD1^G93A^ mutation mice^11^ and TDP43 mice showed the GI symptoms at the early stage of the disease, suggesting the importance to target intestinal functions in the ALS treatment. In 2017, our research paper demonstrated the beneficial role of the microbial metabolite butyrate in the ALS preclinical model.^11^ In the past several years, the novel concepts and the role of gut microbiome and microbial metabolites in ALS pathogenesis have been slowly recognized by the neurology research field. Labarre *et al*. found that the probiotic bacterium *Lacticaseibacillus rhamnosus* HA-114 prevents neurodegeneration in a *C. elegans* model of ALS.^67^ Nicotinamide (Vitamin B3) and nicotinate were found to be reduced in SOD1G93A mice.^5^ Relyvrio (AMX0035), a combination of sodium phenylbutyrate and taurursodiol, has been shown to reduce the rate of decline on a clinical assessment of daily functioning and is associated with longer overall survival.^68^ However, in a large clinical trial, Relyvrio did not work better than a placebo. It has been taken off the US market on April 4, 2024, based on the announcement from Amylyx Pharmaceuticals. Patients with ALS desperately need new therapeutic methods to control the disease. Research on metabolites correlated with the gut microbiome in ALS progression and treatment are still limited and needed.

**Figure 9.**
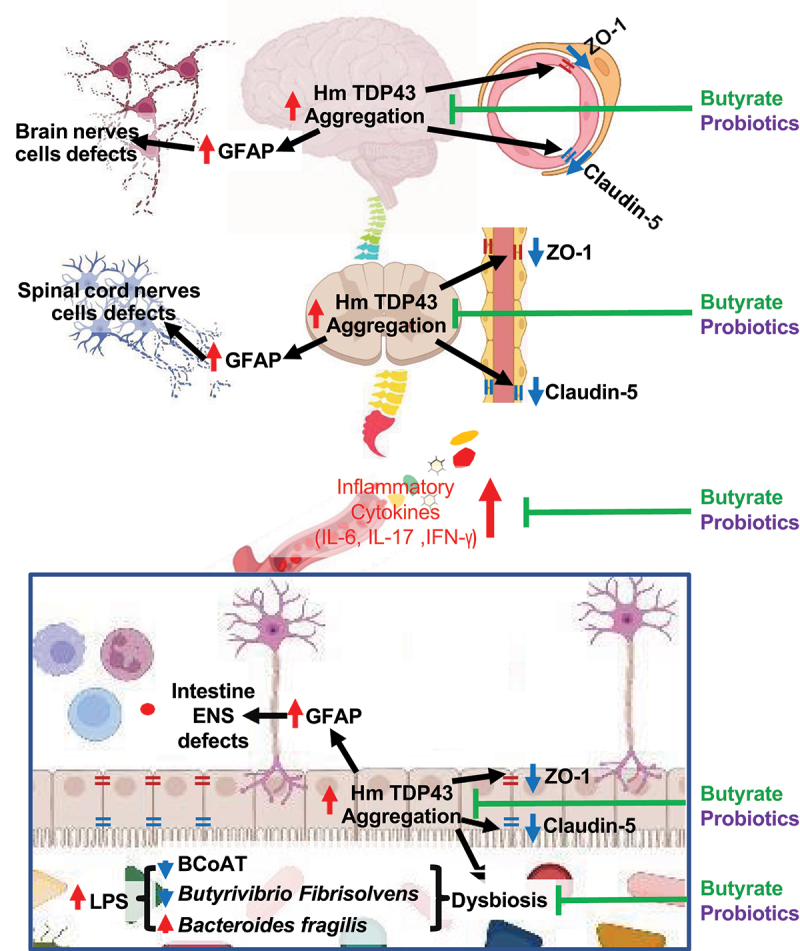
A working model of modulating microbiome in TDP43 mutant mice through the gut-microbiome-brain axis.

In the current study, TDP43 mice treated with butyrate or probiotic VSL#3, intestinal microbial homeostasis was restored, including increased levels of Butyryl-coenzyme A CoA transferase gene, increased butyrate-producing bacteria *Butyrivibrio Fibrisolvens* and decreased *Bacteroides fragilis*, compared to the untreated group. Butyrate is a positive feedback loop among the BCoAT butyrate-producing bacteria, and butyrate supplementation. Previous studies^69,70^ have shown that oral butyrate significantly enhanced the fecal butyrate-producing bacteria pool, evaluated by BCoAT gene. Butyrate is known as a main regulator in the positive feedback loop between the intestinal microbiome and epithelial metabolism. Colonocytes are forced to consume oxygen for β-oxidation of butyrate, the key mechanism of energy generation. The consequent hypoxia in the epithelial cells promotes the growth of obligatory anaerobes, including butyrate-producing bacteria.^70^ VSL#3 is known to enhance intestinal barrier functions in various chronic diseases.^38^ In our current study, we also shown its protective role in enhance BBB in the TDP43 mutant mice. We found the similar protections using butyrate or probiotic VSL#3. As summarized in Figure 9, probiotics and butyrate are able to restore microbiome, reduce serum inflammation, hmTDP43 aggregation and increase barrier functions in the colon, spinal cord, and brain of the TDP43 mutant mice.

The mechanisms of butyrate or probiotics in protecting TDP43 mice are several folds: cleaning protein aggregation, maintaining TJ proteins, inhibiting inflammation, and restoring beneficial bacteria. TDP43 mice had significantly increased hmTDP43 and GFAP, decreased expression of α-SMA, tight junction proteins (ZO-1 and Claudin-5) in the colon, spinal cord, and brain compared with age-matched WT mice, while butyrate or probiotic treatment facilitates the restoration of beneficial microbiome and barrier functions. TDP43 mice showed dysbiosis and high serum inflammation, while butyrate or probiotics treatment can reduce systemic and neuroinflammation.

Microbial metabolites affect the neuron system and muscle cell functions. Previously, we have demonstrated that butyrate treatment are able to restore some of the healthy metabolites in the SOD1^G93A^ ALS mice.^50^ We found changes in carbohydrate levels, amino acid metabolism, and formation of gamma-glutamyl amino acids. The tryptophan signaling is known to regulate host health and link the gut-brain axis.^71,72^ In 13-week-old SOD1^G93A^ mice, we observed significant changes of the tryptophan metabolites 3-indoxysulfate, indoleacetylglycine, and indolepropionylglycine. Declines in gamma-glutamyl amino acids in feces may stem from differential expression of GGT in response to butyrate treatment. Patients with ALS have explored treatment with an amino acid-enriched diet. There was a clinical trial on the tolerability and efficacy of L-Serine in patients with ALS (NCT03580616). In the future study, we would like to learn the changes of metabolites in the TDP43 mice and correlate with the human ALS.

In summary, restoring the intestinal and microbial homeostasis by feeding the TDP43 mice with butyrate or probiotics significantly delayed the disease onset. The mechanisms of the protections are several folds: maintaining TJ proteins, cleaning protein aggregation, and restoring beneficial bacteria. Our study elucidates the critical role of barriers, EGCs, and microbiome in ALS and provides insights into alternative approaches for the disease management.

## Data Availability

The data that support the findings of this study are available from the corresponding author, J.S., upon reasonable request.
